# Genome Sequencing and Pan-Genome Analysis of 23 *Corallococcus spp.* Strains Reveal Unexpected Diversity, With Particular Plasticity of Predatory Gene Sets

**DOI:** 10.3389/fmicb.2018.03187

**Published:** 2018-12-19

**Authors:** Paul G. Livingstone, Russell M. Morphew, David E. Whitworth

**Affiliations:** Institute of Biological Environmental and Rural Sciences, Aberystwyth University, Aberystwyth, United Kingdom

**Keywords:** myxobacteria, comparative genomics, taxonomy, evolution, predator, prey

## Abstract

*Corallococcus* is an abundant genus of predatory soil myxobacteria, containing two species, *C. coralloides* (for which a genome sequence is available) and *C. exiguus*. To investigate the genomic basis of predation, we genome-sequenced 23 *Corallococcus* strains. Genomic similarity metrics grouped the sequenced strains into at least nine distinct genomospecies, divided between two major sub-divisions of the genus, encompassing previously described diversity. The *Corallococcus* pan-genome was found to be open, with strains exhibiting highly individual gene sets. On average, only 30.5% of each strain’s gene set belonged to the core pan-genome, while more than 75% of the accessory pan-genome genes were present in less than four of the 24 genomes. The *Corallococcus* accessory pan-proteome was enriched for the COG functional category “Secondary metabolism,” with each genome containing on average 55 biosynthetic gene clusters (BGCs), of which only 20 belonged to the core pan-genome. Predatory activity was assayed against ten prey microbes and found to be mostly incongruent with phylogeny or BGC complement. Thus, predation seems multifactorial, depending partially on BGC complement, but also on the accessory pan-genome – genes most likely acquired horizontally. These observations encourage further exploration of *Corallococcus* as a source for novel bioactive secondary metabolites and predatory proteins.

## Introduction

The Order Myxococcales (myxobacteria) are well known for their predatory lifestyle, which has been linked to the secretion of antimicrobial secondary metabolites and proteins, within and alongside outer membrane vesicles (Evans et al., 2012; Whitworth et al., 2015; Findlay, 2016; Livingstone et al., 2018). Several myxobacterial metabolites have been purified and shown to be bioactive and therefore myxobacteria represent an underexploited resource for bioactive discovery (Landwehr et al., 2016).

Predatory myxobacteria can be isolated by “baiting” with prey organisms and such approaches have a consistent isolation bias predominantly yielding representatives of just two myxobacterial genera, *Myxococcus* and *Corallococcus* (Livingstone et al., 2017; Mohr et al., 2017). Both genera belong to the family Myxococcaceae, suborder Cystobacterineae, but have received differing research attention. *Myxococcus xanthus* is the single best studied myxobacterium, often taken as the exemplar for many aspects of myxobacterial biology (Whitworth, 2008b; Yang and Higgs, 2014). However, members of the genus *Corallococcus* are comparatively poorly described.

The *Corallococcus* genus currently comprises two validly described species, *C. coralloides* and *C. exiguus*, with *C. macrosporus* having been recently reassigned to the *Myxococcus* genus (Lang and Stackebrandt, 2009; Garcia et al., 2010). In their vegetative phase in the presence of nutrients, they produce colorless swarming growth, while on starvation they form fruiting bodies, which are pale orange or peach colored, horny or coral-shaped, hence the genus name (Garcia and Müller, 2014). Consistent with their predatory activity, several secondary metabolites with potent antibacterial and antifungal activities, such as corallopyronins, coralmycins, and corallorazines, have been characterized from *Corallococcus* spp. (Xiao et al., 2011; Schmitz et al., 2014; Schäberle et al., 2015; Kim et al., 2016).

Nearly twenty years ago, strains formerly designated as *Myxococcus* (Cc e100 and Cc e216) and *Chondrococcus* (Cc c494 and Cc c806) were reclassified into the genus *Corallococcus* and given species status as *C. exiguus* and C. *coralloides*, respectively, based on their 16S rRNA gene sequences (Spröer et al., 1999). The designation of *Corallococcus* as a genus was subsequently supported by biotyping methods such as MALDI-TOF (Stackebrandt et al., 2005), although DNA-DNA reassociation measures and ribotyping hinted at genomic heterogeneity within the genus (Stackebrandt et al., 2007).

16S rRNA sequencing has been widely used as a basis for taxonomic assignment, but the threshold of sequence similarity required for species identification has undergone considerable change (Hagström et al., 2002; Buckley and Roberts, 2007). This has led to suggestions for DNA-DNA hybridization to replace 16S rRNA phylogenetics as the gold standard for the designation of new species (Goris et al., 2007). However, the relative simplicity, low cost and ease of 16S rRNA sequencing has cemented its position as the most frequently used method for species identification in recent years (Chun and Rainey, 2014).

The availability and ever-reducing costs of next generation sequencing in the present decade has allowed whole genome-based methods to be developed for microbial taxonomy. Genome sequences also provide greater insights into an organism’s biology and evolution than 16S rRNA sequences, for instance leading to the concepts of core and accessory genomes from studies into the pan-genomes of taxa (Xiao et al., 2015). Taxonomic studies are consequently moving increasingly from phylogenetic to phylogenomic approaches, highlighting misidentifications and making novel identifications. Many of these identifications are made possible only because of the greater information available from genome level sequencing (Benevides et al., 2017 Tran et al., 2017). Minimal standards have also now been set for using bacterial genomes for taxonomic assignment (Chun et al., 2018).

The predatory activity exhibited by myxobacterial isolates against particular prey species, does not correlate well with 16S rRNA gene sequence based phylogeny (Livingstone et al., 2017). Presumably, this is a consequence of extensive horizontal gene transfer of accessory genes, which has been well-documented for biosynthetic gene clusters (BGCs) such as those involved in microbial competition/predation (Wisecaver and Rokas, 2015). In such a context it is therefore logical to classify taxa by their pan-genome (accounting for lateral and vertical descent) rather than by their 16S rRNA gene sequence (exclusively vertical descent). Understanding a taxon’s pan-genome in this way is required to evaluate the influx of new biosynthetic capabilities into the pan-genome, its “openness,” and therefore to gain an estimate of the untapped metabolic potential of a taxon.

To that end, we describe here the draft genomes of 23 *Corallococcus* isolates, adding substantially to the available sequence data for *Corallococcus*, which previously consisted of a single complete genome. Analysis of the *Corallococcus* genomes suggests that the genus *Corallococcus* comprises at least 9 (8 novel) constituent species, with a large and open pan-genome. We also describe the genomic commonalities and potential for untapped metabolic diversity of the two largest species-groups of *Corallococcus*.

## Materials and Methods

### Cultivation of Strains

All strains used in this study except AB011P were described previously as *Corallococcus* members (Livingstone et al., 2017). AB011P was isolated from soil sampled from farmland at Penrhyncoch near Aberystwyth, using an *Escherichia coli* prey baiting method. Soil samples were placed on nutrient free medium next to a spot of prey organism. Swarming predators which consumed the prey were picked and isolated onto fresh growth media. 16S rRNA sequencing of AB011P and phylogenetic analysis, according to the method described by Livingstone et al. (2017) confirmed AB011P belonged to the genus *Corallococcus*. All bacterial strains (including AB011P) were cultivated as described previously (Livingstone et al., 2017) and their predatory activity was assayed against a panel of prey organisms. Myxobacteria were spotted onto lawns of each prey and the resulting zone of prey killing measured, exactly as described by Livingstone et al. (2017), except killing zones were measured after 7 days instead of 4 days in order to increase the sensitivity of the assay.

### Genome Sequencing

Strains of *Corallococcus* spp. were sequenced using 2 × 250 bp paired-end reads on an Illumina Hiseq 2500 platform by either the Centre for Genomic Research (CGR Liverpool, United Kingdom – strains AB011P and AB038B) or MicrobesNG (Birmingham, United Kingdom – another 21 strains). MicrobesNG were provided with cell masses, while DNA was extracted from AB011P and AB038B using the DNeasy Blood and Tissue kit (Qiagen) and quantified using a nanodrop spectrophotometer before provision to the CGR. Kraken 2 was used to identify the closest reference genomes for read mapping (Wood and Salzberg, 2014). BWA-MEM was used to check the quality of the reads (Li and Durbin, 2009) while *de novo* assembly was performed using SPAdes 3.7 (Bankevich et al., 2012). Annotation was undertaken using Prokka 1.1 (Seemann, 2014), and RAST 2.0 (Aziz et al., 2008) and contigs with coverage less than 7-fold were removed. The genome sequence of *Corallococcus coralloides* DSM2259^T^ was retrieved from the NCBI database. Genomes sequenced in this study are listed in Table 1 and available from the NCBI nucleotides database under accession numbers RAWS00000000 (AB004), RAVX00000000 (AB011P), RAWR00000000 (AB018), RAWQ00000000 (AB030), RAWP00000000 (AB032C), RAWO00000000 (AB038B), RAVW00000000 (AB043A), RAWN00000000 (AB045), RAWM00000000 (AB047A), RAWL00000000 (AB049A), RAWK00000000 (AB050A), RAWJ00000000 (AB050B), RAWI00000000 (CA031B), RAWH00000000 (CA031C), RAWG00000000 (CA040B), RAWF00000000 (CA041A), RAWE00000000 (CA043D), RAWD00000000 (CA047B), RAWC00000000 (CA049B), RAWB00000000 (CA051B), RAWA00000000 (CA053C), RAVZ00000000 (CA054A), and RAVY00000000 (CA054B).

**Table 1:** General features of the 23 *Corallococcus* draft genomes compared to the complete *C. coralloides* DSM 2259^T^ genome (Huntley et al., 2012).

Strains	Size (bp)	%GC	Coding sequences	N50	L50	Mean coverage	Group
DSM2259	10,080,619	69.9	8227				A
AB004	10,604,304	69.4	8407	26897	119	35.2	A
AB011P	10,177,911	69.8	8259	279277	11	28.9	A
AB018	10,463,000	69.4	8390	37048	94	49.2	A
AB030	10,640,049	69.6	8573	37630	88	55.0	A
AB032C	10,451,389	69.5	8335	73098	43	127.0	A
AB038B	10,785,584	69.2	8559	431941	7	26.3	A
AB043A	10,150,784	70.3	8185	19246	164	40.8	A
AB045	9,940,502	69.9	8035	37416	80	76.2	A
AB047A	9,538,803	69.8	7720	54870	51	164.7	A
AB049A	9,526,569	69.7	7638	17173	170	33.6	A
AB050A	9,983,374	70.0	8007	29422	115	56.9	A
AB050B	9,397,643	70.1	7683	42207	68	99.2	A
CA031B	10,514,033	69.6	8139	20084	152	74.2	B
CA031C	10,231,016	69.9	8011	30964	97	33.8	B
CA040B	10,401,616	70.2	8043	26404	119	118.2	B
CA041A	10,265,543	69.5	8203	30701	110	82.3	A
CA043D	10,794,417	69.9	8724	37418	80	67.3	A
CA047B	10,336,837	69.9	8133	25434	123	81.4	B
CA049B	9,633,170	70.2	7779	34676	82	37.2	A
CA051B	10,527,286	70.3	8250	15085	220	30.6	B
CA053C	10,518,560	70.1	8360	20162	153	54.2	B
CA054A	10,352,759	69.5	8160	24469	134	66.8	B
CA054B	9,916,432	70.0	8038	39232	80	47.8	A


*Strains were assigned to Groups A and B based on their sequence similarity and phylogenetic relationships – see the text for details.*

### Phylogenetic Analyses

Complete 16S rRNA gene sequences were obtained from sequenced genomes and used to query a BLASTn search of the NCBI database to identify their closest relatives. Query sequences along with those from relatives with >92% similarity and >82% nucleotide coverage were aligned and a neighbor-joining tree was constructed in MEGA-7.0 (Kumar et al., 2016), using the Kimura 2-parameter model with 500 bootstrap replicates. AMPHORA2 was used to predict phylogenetic relationships based on 31 concatenated and aligned marker genes (Kerepesi et al., 2014), and a maximum likelihood tree was generated using a Jones-Taylor-Thornton model on MEGA 7.0 (Kumar et al., 2016). We also used PhyloPhlAn to provide a more highly resolved phylogeny, based on 400 conserved genes (Segata et al., 2013). Newick format tree files were uploaded onto iTOL, a web based tool for annotating and editing trees (Letunic and Bork, 2016).

### Genome Similarity Measures

The average nucleotide identity (ANI) is a widely accepted genome-based method for species delineation and an ANI-based all-vs-all matrix and resulting clustering tree were constructed using the ANI-Matrix genome-based distance matrix calculator (Rodriguez and Konstantinidis, 2016). The digital-DNA/DNA hybridization (DDH) was calculated for pairs of genomes using the genome-to-genome distance calculator (GGDC 2.1) (Meier-Kolthoff et al., 2013). Results from GGDC Formula 2 are presented, as Formula 2 is the most robust Formula when applied to incomplete genomes (Auch et al., 2010).

### Pan-Genome Analysis

Orthogroups (sets of orthologs) were compiled and trees based on orthogroup member presence/absence were constructed both using OrthoFinder 1.1.8 (Emms and Kelly, 2015). Pan-genome analysis was carried out using Roary (Page et al., 2015). Phandango (Hadfield et al., 2017) and recommended R scripts were used to view the resulting output graphs. Using RAxML (Stamatakis et al., 2004) core genome gene sequences were aligned and maximum likelihood trees produced for viewing and editing using iTOL (Letunic and Bork, 2016).

### Functional Annotation

SPINE (Ozer et al., 2014) was used to separate core and accessory gene sequences. Initially genomes were aligned using the NUCmer function of the integrated MUMmer software package to identify core genes. Core gene sequences were then mapped to individual genomes using AGEnt (Ozer et al., 2014), allowing identification of the accessory (non-core) genes sequences in each genome. Accessory gene sequences were then clustered using ClustAGE (Ozer et al., 2014). Core and accessory gene sequences were then functionally annotated using eggNOG mapper and antiSMASH (Weber et al., 2015; Huerta-Cepas et al., 2017).

## Results

### Genome Sequencing of 23 *Corallococcus* Strains

Only two “genomes” assigned to *Corallococcus* spp. were publically available at the time of this study, including the complete 10.08 Mbp genome of the type strain *C. coralloides* DSM 2259^T^ (Huntley et al., 2012). The second “genome” was a 1.56 Mbp contig assembled from metagenomic data, which we excluded from further comparisons. In a previous study, we isolated a large number of strains, identified as *Corallococcus* spp., and characterized their predatory activity (Livingstone et al., 2017). Twenty two of those *Corallococcus* strains, with diverse predatory profiles, and a novel *Corallococcus* spp. isolate (AB011P), with broad and potent antimicrobial activity, were chosen for genome sequencing. All strains grew well on VY/2 Agar and were sequenced variously by the Centre for Genomic Research (Liverpool, United Kingdom – strains AB011P and AB038B) and MicrobesNG (Birmingham, United Kingdom – 21 strains), using the Illumina Hi-Seq platform.

The number of contigs in each draft genome ranged from 104 to 1,546, with an average of 716 per genome. The L50 values (the largest *X* contigs which together constituted more than half of the genome sequence) had a median of 93, with an average N50 value (the size of the *X*th contig) of 60,472 bp (Table 1). Mean sequence coverage was greater than 25-fold for all genomes (averaging 65-fold coverage), with a mean %GC content of 69.9. Assembled draft genomes were consistently large, having an average size of 10.22 Mbp, with the smallest genome (strain AB050B) being 9.40 Mbp and the largest (strain CA043D) being 10.79 Mbp (Table 1). Consequently, the number of coding sequences (CDS) also did not vary widely, with an average of 8,158, ranging between 7,638 and 8,724. Genome sequence data for this project have been deposited in the NCBI nucleotide database.

### Phylogenetic Analysis of *Corallococcus* spp. Reveals Major Subdivisions

A phylogenetic tree (Supplementary Figure S1) was constructed using 16S rRNA gene sequences retrieved from the 23 genome sequenced strains, 37 *Corallococcus* spp. DSM strains and 8 non-*Corallococcus* myxobacterial strains from the NCBI database (using *Polyangium cellulosum* as the outgroup). All the non-*Corallococcus* sequences grouped together, separately from 58 of the 60 *Corallococcus* sequences. Two of the DSM *Corallococcus* sequences (*C. coralloides* DSM 52496 and C. *coralloides* DSM 52498) grouped into a clade with *Myxococcus*/*Pyxidicoccus* and querying them against the NCBI 16S database gave best hits to *Myxococcus fulvus*, suggesting they had been misclassified as *Corallococcus* members. The remaining *Corallococcus* sequences grouped together, with strong (86%) bootstrap support (Supplementary Figure S1). There was little difference between the placement of the type strains of *C. coralloides* (DSM 2259^T^) and *C. exiguus* (DSM 14696^T^), as expected given they are likely conspecifics (Garcia et al., 2010).

**FIGURE 1:**
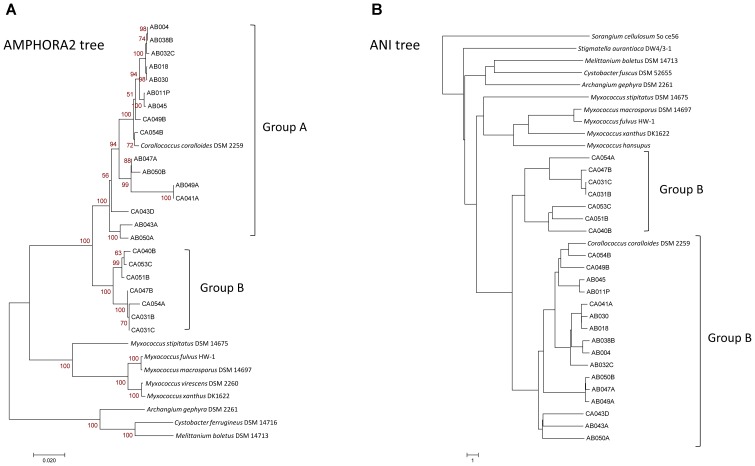
Phylogenetic trees of *Corallococcus* isolates and other genome-sequenced myxobacteria. **(A)** AMPHORA2 tree based on 31 conserved genes. **(B)** Tree based on relative distance calculated from the genomic ANI (average nucleotide identity).

Trees were also constructed for sequenced organisms using AMPHORA2 on 31 conserved genes (Figure 1A), based on genomic nucleotide identity (Figure 1B), using PhyloPhlAn on 400 conserved genes (Supplementary Figure S2), using RAxML on core pan-genome genes, and based on the presence/absence of orthogroup members (Supplementary Figure S3). All trees had similar branching patterns, and *Corallococcus* genomes consistently grouped into two major clades. The first, and the larger clade, containing *Corallococcus coralloides/exiguus*, designated as the Group A *Corallococci* and the second smaller clade designated as the Group B *Corallococci*. From inspecting the 16S sequence tree, (Supplementary Figure S1), the following DSM strains are members of the Group B *Corallococci*: DSM 51298, DSM 51619, DSM 51620, DSM 51625, DSM 51639, DSM 51643, DSM 52500, and DSM 52501.

### Genomic Metrics Suggest *Corallococcus* Comprises at Least Nine Discrete Species

Average nucleotide identity and digital DNA-DNA hybridization (dDDH) values, as calculated using the genome-to-genome calculator (GGDC), are being increasingly used to benchmark genomospeciation. Currently an ANI of ≥95% is required to conclude that two organisms belong to the same species (Richter and Rosselló-Móra, 2009; Sangal et al., 2016). An ANI of ≤92% is taken as evidence that two genomes belong to different species (<75% for different genera), while pairs of genomes giving intermediate ANI values (93–94%), likely belong to separate sub-species (Sangal et al., 2016).

ANI values for all pair-wise comparisons of the 24 *Corallococcus* genomes are presented in Table 2. All pair-wise comparisons gave ANI values ≥82%, confirming *Corallococcus* is a single genus, although Group A and Group B may represent discrete sub-genera. Using a ≤92% ANI cut-off, nine “species” groups of genomes can be observed, with members from different groups belonging to separate species (Table 2). Applying an ANI value cut-off of ≥95% gives 11 groups of genomes, within which members definitely belong to the same species. However, pair-wise comparisons giving ANI values of 93–94% associated four of those 11 into two larger groups, suggesting that two of the species groups each contain two discrete sub-species.

**Table 2:** Average Nucleotide % Identity (ANI) and Digital DNA-DNA Hybridisation (dDDH) comparisons between all sequenced *Corallococcus* genomes.

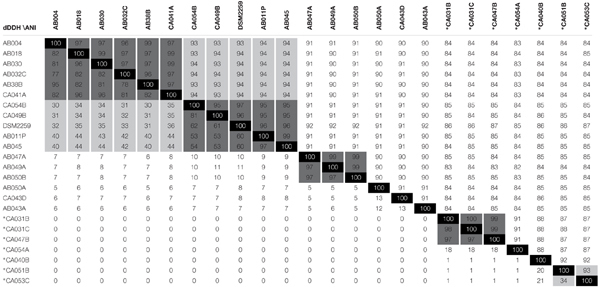

*ANI values are presented above the diagonal, with dDDH values below. Values shaded light gray have ANI integer values between 93 and 94% or dDDH integer values between 30 and 50%, while values shaded dark gray have ANI values between 95 and 100% or dDDH values between 50 and 100%. Strains marked with an asterisk (^∗^) belong to Group B, others belong to Group A.*

Table 2 also provides the dDDH values for pair-wise comparisons of the *Corallococcus* genomes. The use of dDDH values for species delineation is not well benchmarked, however, cut-offs of >50 and >30% give species groups mirroring the results of ANI-based species definitions at ANI values of >95 and >93%, with 9 and 11 species groups, respectively. A dDDH cut-off of > 70% has been proposed by Richter and Rosselló-Móra (2009) to define membership of the same species. Applying a >70% cut-off gives 13 species groups, due to splitting the Group A subspecies containing *C. coralloides* DSM 2259^T^ into three separate species, which is not supported by ANI values (>95%).

From the ANI and dDDH values, we therefore propose conservatively that the genus *Corallococcus* comprises at least nine species, with five species in Group A and four in group B: Group A species 1 subspecies 1 (CA049B, CA054B, DSM2259^T^, AB011P, and AB045) and subspecies 2 (AB004, AB018, AB030, AB032C, AB38B, and CA041A), Group A species 2 (AB047A, AB049A, and AB050B), Group A species 3 (AB043A), Group A species 4 (AB050A), Group A species 5 (CA043D), Group B species 1 subspecies 1 (CA051B) and subspecies 2 (CA053C), Group B species 2 (CA040B), Group B species 3 (CA054A), and Group B species 4 (CA031B, CA031C, and CA047B).

### The *Corallococcus* Pan-Genome Is Large and Open

To analyze the *Corallococcus* pan-genome in detail, Roary was used (with a 90% BLASTp percentage identity cut-off) to cluster the genes encoding complete protein sequences into core (hard core and soft core) and accessory (shell and cloud) genomes (Page et al., 2015). Hard core genes are found in >99% genomes, soft core genes are found in 95–99% of genomes, shell genes are found in 15–95%, while cloud genes are present in less than 15% of genomes. Of 43,561 orthologous proteins, the *Corallococcus* pan-genome core comprised 2,486 genes (on average 30.5% of each genome), with the accessory genome containing 9,846 genes in the shell (22.6%) and 31,229 in the cloud (71.7%) (Table 3). Enough genomes were available in Group A and Group B to also undertake pan-genome analysis of the two groups separately (Table 3). As expected, due the smaller number of genomes in Groups A and B, they showed larger cores and smaller accessory pan-genomes than for the whole set of *Corallococcus* genomes. Surprisingly the Group A core pan-genome was substantially larger than that of Group B (4,195 and 3,282, respectively), despite including more than twice as many genomes (17 and 7, respectively), with each Group A member having a smaller complement of accessory genes than Group B.

**Table 3:** The pan-genomes of *Corallococcus*, Group A *Corallococci*, Group B *Corallococci*, and *Myxococcus*.

	*Corallococcus* spp.	Group A *Corallococci*	Group B *Corallococci*	*Myxococcus* spp.
Genomes (*n*)	24	17	7	6
# discrete species	9–11	5–6	4–5	6
Mean genes per genome	8,161	8,162	8,157	7,286
Pan-genome size (genes)	43,561	26,491	21,292	23,833
Average % of genes in the core	30.5%	51.4%	40.2%	8.4%
Core genes (total) (%)	2,486 (5.7%)	4,195 (15.8%)	3,282 (15.4%)	609 (2.6%)
-Core genes (hard) (%)	-2,315 (5.3%)	-4,195 (15.8%)	-3,282 (15.4%)	-609 (2.6%)
-Core genes (soft) (%)	-171 (0.4%)	-0 (0%)	-0 (0%)	-0 (0%)
Accessory genes (total) (%)	41,075 (94.3%)	22,296 (84.2%)	18,010 (84.6%)	23,224 (97.4%)
-Accessory genes (shell) (%)	-9,846 (22.6.0%)	-7,471 (28.2%)	-8,009 (37.6%)	-23,224 (97.4%)
-Accessory genes (cloud) (%)	-31,229 (71.7%)	-14,825 (56.0%)	-10,001 (47.0%)	-0 (0%)
Extrapolated pan-genome (new genes) when *n* = 101	101,987 (555)	55,378 (228)	77,244 (372)	121,349 (747)
Extrapolated pan-genome (new genes) when *n* = 501	245,333 (269)	107,520 (89)	167,900 (163)	327,798 (406)
Extrapolated pan-genome (new genes) when *n* = 1001	358,522 (196)	143,228 (59)	234,844 (114)	503,647 (312)
Extrapolated core genes at *n* = ∞	988 (39.7%)	4,058 (96.7%)	3,105 (94.6%)	405 (66.5%)
COGs enriched in core genome	E F H J	E F I	F J	E F J
COGs enriched in accessory genome	Q V	L Q V	Q	P Q
BGCs for named metabolites – core	5 (10.0%)	5 (15.6%)	6 (18.2%)	7 (26.9%)
BGCs for named metabolites – accessory	45 (90.0%)	27 (84.4%)	27 (81.8%)	19 (73.1%)


*For each pan-genome, the composition is presented, together with extrapolations to larger numbers of included genomes, and the number of biosynthetic gene clusters (BGCs) with similarity to those producing named compounds. COG categories enriched in particular sub-pan-genomes are: E, amino acid transport and metabolism; F, nucleotide transport and metabolism; H, coenzyme transport and metabolism; I, lipid transport and metabolism; J, translation, ribosomal structure and biogenesis; L, replication, recombination and repair; P, inorganic ion transport and metabolism; Q, secondary metabolites biosynthesis, transport, and catabolism; and V, defense mechanisms.*

**FIGURE 2:**
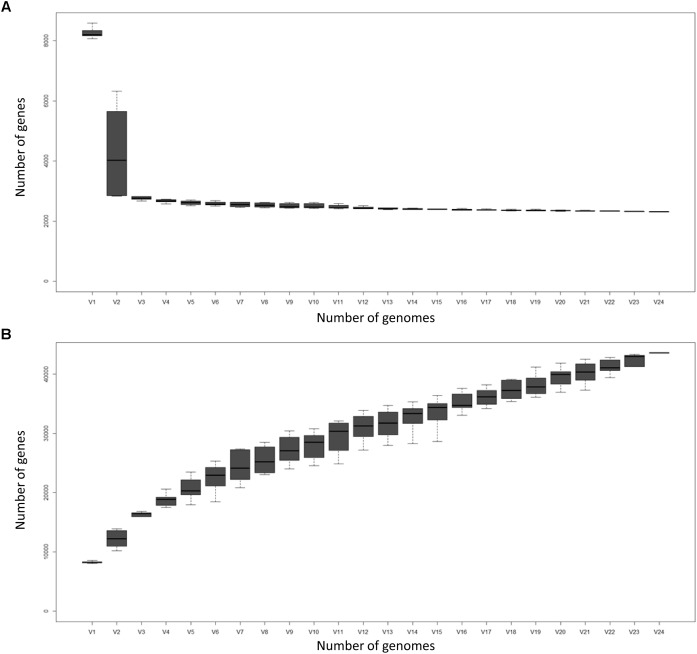
The *Corallococcus* core genome **(A)** and pan-genome **(B)** as a function of the number of genomes included (V1–V24). Boxes represent +/- one standard deviation around the median number of genes while whiskers indicate +/- two standard deviations.

Figure 2 displays the number of core genes (A) and the pan-genome size (B) as a function of the number of included genomes. The number of core genes plateaus and if the number of core genes is plotted against the reciprocal of the number of genomes included, the size of the core pan-genome at an infinite number of genomes would be expected to be 988, suggesting that 1498 (60.3%) of the current core genes will prove to actually be accessory genes. However, such conclusions should be taken with caution, as 100+ genome sequences should ideally be used for these types of comparisons (Whitworth, 2008a). Nevertheless, the core pan-genome at infinite genomes is much higher for Groups A and B (96.7 and 94.6% of the current core, respectively), presumably as a consequence of the greater sequence similarity of orthologs within the groups compared to between the groups.

The size of the pan-genome increases steadily with the addition of each further genome suggesting *Corallococcus* has a large, open pan-genome. By fitting the curve in Figure 2A to a power law (8127n^0.5481^) it is possible to extrapolate to n genomes (Table 3). Even when 1,000 *Corallococcus* genomes have been sequenced, it is expected that sequencing another one would add a further 196 genes to the pan-genome. Only when ∼4,500 genomes have been sequenced would fewer than 100 novel genes be identified per further genome sequenced.

In comparison to the *Corallococcus* pan-genome, the *Myxococcus* pan-genome (which included genomes of five validly described species [*fulvus* HW1, *macrosporus* DSM 14697, *stipitatus* DSM 14675, *virescens* DSM 2260, and *xanthus* DK1622] and *M. hansupus*), (Supplementary File S1) had a substantially smaller core genome (609 genes) and a proportionately larger cloud/shell pan-genome (91.6% of each genome).

### The *Corallococcus* Accessory Pan-Proteome Is Enhanced for Conflict/Predation

SPINE (Ozer et al., 2014) was used to separate core from accessory gene sequences, and the resulting protein sets annotated using eggNOG (Supplementary File S1), for all 24 *Corallococcus* spp., Group A *Corallococci* and Group B *Corallococci*. The percentage abundances of COG functional categories within a set were calculated and compared between protein sets to obtain a fold-difference in relative abundance. Inclusion criteria of log_2_(relative abundance) > ±1, with an abundance > 2%, were applied to identify COG categories that were relatively enriched or impoverished in a protein set. There were no clear differences in the COG categorizations of the core pan-proteomes of Group A, Group B or all *Corallococci*. However, differences between the core and accessory pan-proteomes were observed in categories relating to conflict/predation (Table 3). The accessory pan-proteomes of Group A, Group B and all *Corallococci* were enriched for category Q (Secondary metabolites biosynthesis, transport, and catabolism) compared to their respective core pan-proteomes, while the Group A *Corallococci* and all *Corallococci* (but not Group B *Corallococci*) accessory pan-proteomes were also enriched for proteins of category V (Defense mechanisms). As expected the core pan-proteomes were variously enriched for housekeeping COG functions E (Amino acid transport and metabolism), F (Nucleotide transport and metabolism) and J (Translation, ribosomal structure and biogenesis).

Similarly to *Corallococcus*, the *Myxococcus* accessory pan-proteome (Supplementary File S1) was enriched in proteins assigned to COG category Q (secondary metabolism) compared to the core proteome. However, in contrast to *Corallococcus*, the *Myxococcus* core pan-proteome was relatively enriched in proteins of COGs J (Translation, ribosomal structure, and biogenesis) and U (Intracellular trafficking, secretion, and vesicular transport) and relatively impoverished in proteins of COGs G (Carbohydrate transport and metabolism) and P (Inorganic ion transport and metabolism).

### *Corallococcus* Genomes Have Substantial and Diverse Biosynthetic Potential

AntiSMASH prediction of BGCs (Supplementary File S2) revealed that the average *Corallococcus* genome possesses 55 BGCs (1315 BGCs were found amongst the 24 genomes). CA051B had the highest number of BGCs (94), while the type strain *C. coralloides* DSM 2259^T^ had the lowest (35). Non-ribosomal peptide synthetases (NRPS) were the most prevalent BGCs (21 per genome on average), but there were also on average ten T1PKS-NRPS and five bacteriocin gene clusters per genome. Amongst these genomes, in four cases a single genome contained a BGC for a unique predicted compound class (AB043A is predicted to produce a lantipeptide-β-lactam, AB047A makes a NRPS-T1PKS-linaridin molecule, CA049B makes a bacteriocin-T1PKS-NRPS hybrid, while CA054A produces a nucleoside).

Many of the *Corallococcus* BGCs showed significant similarity to BGCs producing known “named” metabolites (Supplementary File S2). Amongst the 24 genomes, BGCs producing 50 named metabolites/classes were predicted. Five predicted “core” named metabolite BGCs were present in at least 23 of the 24 genomes (including alkylresorcinol, geosmin, and nostopeptolide), 25 “cloud” named BGCs were present in three or fewer genomes (including althiomycin, chivosazole, crocacin, cryptophycin, cyanopeptin, nannoystin, pellasoren, streptomycin, teixobactin, and thanamycin), while the other 20 “shell” named metabolite BGCs were present in between four and 22 genomes each. Group A and group B *Corallococci* also had a small core set of BGCs producing named metabolites (five and six, respectively). However, they were different in character between the two groups. Leupyrrin was predicted to be produced by each member of Group B, but was not predicted for any member of Group A, making the leupyrrin BGC a distinctive feature of Group B genomes.

The pattern of a small set of core BGCs and a large repertoire of accessory BGCs was also observed for BGCs predicted to produce unnamed, and thus potentially novel, metabolites. SPINE was used to separate core from accessory genes for the *Corallococcus* genomes, and antiSMASH predictions generated. There were twenty BGCs within the core pan-genome (including those producing four named metabolites) and 290 BGCs within the accessory pan-genome (including 42 predicted to produce 21 different named metabolites). Thus, named metabolites only represent a small proportion of both the core and accessory secondary metabolomes, and the core metabolome is only a small fraction of the metabolic diversity found across the pan-genome.

These trends were also observed for the *Myxococcus* secondary metabolome. AntiSMASH predictions of individual *Myxococcus* genomes revealed BGCs predicted to produce seven “core” named compounds (alkylresorcinol, carotenoid, dkxanthene, geosmin, myxoprincomide, puwainaphycin, and VEPE_/_AEPE_/_TG-1) in all six species. In addition, a further 37 BGCs were predicted to produce another 19 “accessory” named products. Using SPINE to split the core and accessory pan-proteomes gave just one BGC in the core (producing VEPE_/_AEPE_/_TG-1) and 184 BGCs in the accessory genome including 47 predicted to make 26 different named metabolites.

### Predation Correlates Poorly With Taxonomy and BGC Complement

To investigate relationships between taxonomy, BGCs and predation, the predatory activity of all strains was assayed against 10 prey organisms, including yeast, Gram-negative bacteria and Gram-positive bacteria. *Corallococcus* strains were inoculated onto lawns of potential prey organisms and the diameter of the zone of killing was measured after incubating for 7 days (Supplementary File S3). The pattern of predatory activity and prey susceptibility was similar to previous descriptions with *Corallococcus* strains exhibiting broad but patchy activity against all prey (Livingstone et al., 2017).

**FIGURE 3:**
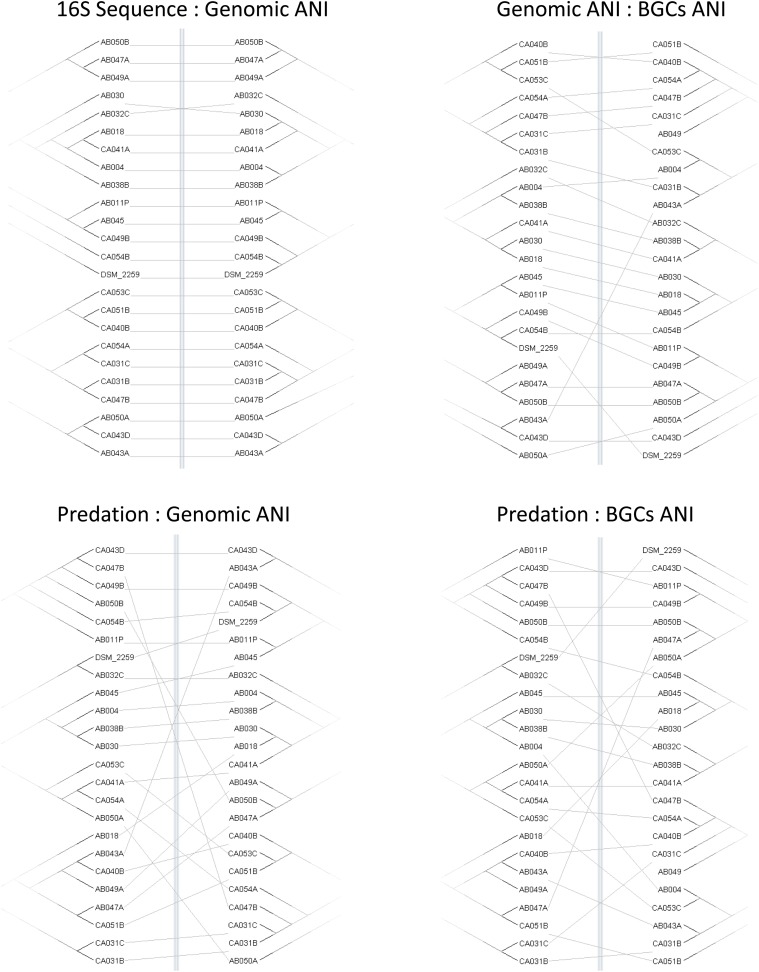
Congruence of taxonomy, BGC, and predation trees. Tanglegrams show pairs of trees reordered for maximal alignment. Trees were variously constructed from the matrix of predatory assay data (Predation), 16S rRNA gene sequences (16S Sequence), genome-wide average nucleotide identity (Genomic ANI), and the average nucleotide identity of biosynthetic gene clusters (BGCs ANI).

Four trees (predation, ANI, 16S, and BGCs) were constructed based on the profiles of predatory activity (Supplementary File S3), genomic ANI (Table 2), 16S rRNA sequence similarity and ANI trees based on the DNA sequences of the BGCs from each strain, extracted from antiSMASH predictions. Differences in matrix structure for sequence similarity and predatory activity precluded generation of correlation coefficients. Therefore, similarities and differences between the trees were visualized as tanglegrams (Figure 3) where the branches of pairs of trees are rearranged to give maximum similarity in topology. Tanglegrams revealed the greatest congruence between taxonomy (both ANI and 16S) and BGC trees with the predation tree exhibiting much less congruence with either taxonomy or BGC trees. Our interpretation of this result is that predatory activity is not solely dependent on BGCs or non-BGC genes acquired by vertical descent (captured in the ANI). Therefore, genes acquired by horizontal gene transfer are likely to be important determinants of predatory activity against particular prey.

## Discussion

### Genome-Based Taxonomy Suggests at Least Nine Species Within *Corallococcus*

In studies isolating predatory myxobacteria from soil, strains belonging to the *Corallococcus* genus commonly predominate, often representing more than 70% of isolates (Livingstone et al., 2017; Mohr et al., 2017). Their colorless swarms are easily distinguished from the other most abundant myxobacterial genus, *Myxococcus*, which is brightly pigmented yellow/orange. Currently, two species of *Corallococcus* are recognized, *C. exiguus* and *C. coralloides*. Although diversity within the genus has been acknowledged (Spröer et al., 1999; Stackebrandt et al., 2007), it seems likely that the two type strains belong to a single group. Garcia et al. (2010), argue that *C. exiguus* and *C. coralloides* are the same species which is also supported from the 16S inferred phylogeny of the current work.

Traditionally, a polyphasic approach has been adopted for the identification and classification of myxobacteria, based on biochemical markers, cell/population physiology, and sequence data. However, with the recent explosion of sequence information, there is increasing acceptance of using sequence data as a basis for taxonomic assignment with whole genome sequence data seen as more reliable and with greater discriminatory power than 16S rRNA gene sequence analysis alone (Janda and Abbott, 2007; Richter and Rosselló-Móra, 2009). Increasingly, genome-based studies are revealing misidentifications and misclassifications of bacteria, highlighting problems arising from single-gene 16S rRNA phylogeny (Benevides et al., 2017; Tran et al., 2017).

Recent studies have proposed minimum standards for species identification based on an overall genome related index (OGRI) which incorporates dDDH and ANI values (Chun et al., 2018). When using the recommended thresholds for ANI and dDDH, the 23 *Corallococcus* genomes sequenced in the current work fall into at least nine genomospecies with potentially as many as 11 identified. Further biochemical and phenotypic analyses are thus required to propose members of these genomospecies as type strains for novel species of *Corallococcus*. Nevertheless, identifying 8 candidate novel species from amongst 23 novel isolates implies a huge diversity of *Corallococcus* in the environment.

### The Pangenome of *Corallococcus* Is Open

Across the full set of 24 *Corallococcus* genomes, the average genome is around one-third core and two-thirds accessory genome, in line with other bacterial species and genera (Lapierre and Gogarten, 2009). For bacteria, the “average” organism has a core pan-genome of around 1650 genes when calculated within a species, reducing to ∼1,350 genes when analyzed across a genus (Vernikos et al., 2015). However, this core pan-genome will depend on the percentage identity cut-offs used, the numbers of genomes included and any sampling biases within the various taxa. *Corallococcus* genomes are large (around 10 Mbp) and the *Corallococcus* core pan-genome is therefore proportionately large (around 2,500 genes). For *Corallococcus*, three-quarters (76.0%) of the accessory genome was composed of cloud genes being identified in three or fewer of the 24 genomes.

It is apparent that *Myxococcus* has access to a larger pool of accessory genes than *Corallococcus* and that *Corallococcus* has a larger set of “core” functions. However, the relative diversity in the *Myxococcus* pan-genome could be a consequence of including single representatives from each *Myxococcus* species. Thus, it would be interesting to characterize the *Myxococcus* pan-genome if generated from 20 independently isolated wild-type strains.

### The Genomics of Predation

Myxobacteria have been actively screened for natural products for several decades, with more than 100 core structures and over 500 derivatives now having been published (Herrmann et al., 2017). Secondary metabolites with potent antimicrobial properties have been obtained from *Corallococcus* cultures, including corallopyronin, corallorazine, and coralmycin (Schmitz et al., 2014; Schäberle et al., 2015; Kim et al., 2016). However, the analysis of *Corallococcus* genome sequences for BGCs with the potential to synthesize novel metabolites (Xiao et al., 2011) has not previously been undertaken.

Phylogenetic trees based on the sequences of *Corallococcus* BGCs are only partially similar to those based on *Corallococcus* genomic sequence similarity (Figure 3), implying gene gain or loss has a substantial impact on the complement of BGCs in a strain’s genome. This suggestion was supported by *Corallococcus* pan-genome analysis, which showed that only a small proportion of the BGCs in the pan-genome could be considered core. Of the 50 BGCs with similarity to those producing named compounds, fully half were found in three or fewer of the 24 genomes. This implies that the full pan-genome of *Corallococcus* will include a huge array of BGCs. Therefore, it will be interesting to determine to what extent the *Corallococcus* and *Myxococcus* pan-genomes share pools of BGCs and whether there are “*Corallococcus*-exclusive” BGCs awaiting discovery.

It also seems likely that non-BGC horizontally acquired genes will prove to be important determinants of predatory activity given that trees based on predatory activity were barely congruent with those based on genomic ANI (Figure 3). Individual strains of *Corallococcus* exhibit very different patterns of predatory activity against different prey species (Supplementary File S3), and only around 30% of any *Corallococcus* strain’s gene set is shared by all other *Corallococcus* strains (Table 3). Presumably, some recently acquired genes confer selective advantages during predation, or conversely, loss of predatory genes might have impaired a strain’s ability to predate. It is an important goal to identify such genes and to discover the mechanisms by which those genes affect predatory activity. To that end, making available 23 genome sequences, from at least nine species of *Corallococcus*, should prove to be an important resource for the continued genetic exploration and exploitation of these inherently antimicrobial bacteria.

## Author Contributions

PL and DW devised the research and analyzed the data. PL undertook the experimental work and data generation. DW and RM supervised the project. PL and DW wrote the manuscript, and all authors edited and approved the submitted manuscript.

## Conflict of Interest Statement

The authors declare that the research was conducted in the absence of any commercial or financial relationships that could be construed as a potential conflict of interest.

## Supplementary Material

Figure S1 16S rRNA gene sequence tree of newly sequenced and DSM *Corallococcus* strains, with other myxobacteria included as an outgroup.

Figure S2 PhyloPhlAn phylogenetic tree of 400 gene sequences from the 24 genome-sequenced *Corallococcus* strains.

Figure S3 OrthoFinder tree based on the presence/absence matrix of orthogroups amongst the 24 sequenced *Corallococcus* genomes.

FILE S1 EggNOG annotations of core and accessory genes for all 24 *Corallococci*, Group A *Corallococci*, Group B *Corallococci* and *Myxococcus* genomes. Sequences outputted from SPINE and used as inputs into EggNOG are also provided as separate rows in a further worksheet.

FILE S2 AntiSMASH predicted compound classes by genome and named compounds predicted to be synthesized by genome.

FILE S3 Predation activity of *Corallococcus* strains against ten prey organisms (predation zone diameter 7 days after inoculation).
