# Supplementary material for: Genome Sequencing and Pan-Genome Analysis of 23 Corallococcus spp. Strains Reveal Unexpected Diversity, With Particular Plasticity of Predatory Gene Sets
Source: Front Microbiol. 2018 Dec 19;9:3187. doi: 10.3389/fmicb.2018.03187 (PMC6306037; doi:10.3389/fmicb.2018.03187)
Supplement: Figure S1 — 16S rRNA gene sequence tree of newly sequenced and DSM Corallococcus strains, with other myxobacteria included as an outgroup. [file Presentation_1.PPTX]

## Slide 1
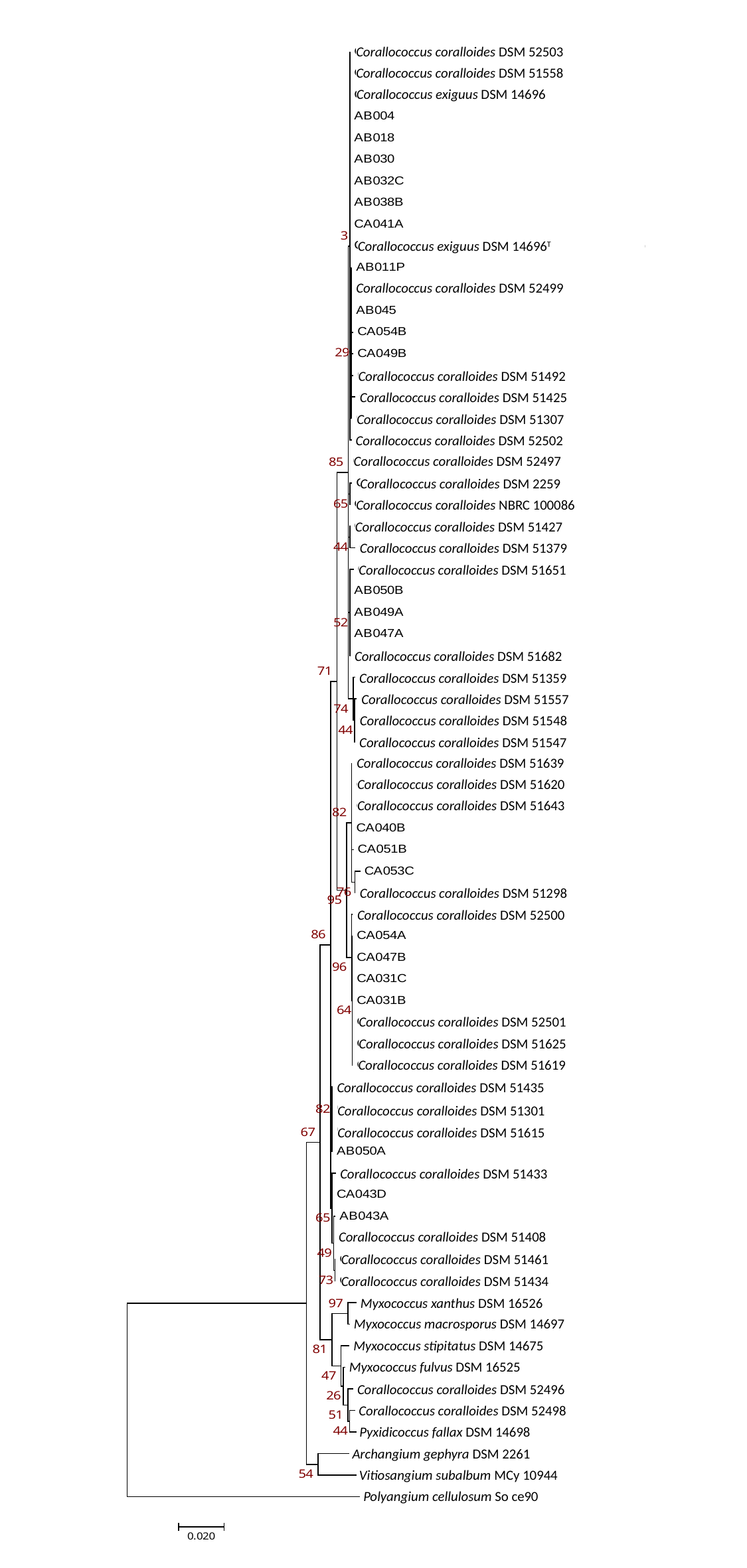

Corallococcus coralloides DSM 52503
Corallococcus coralloides DSM 51558
Corallococcus exiguus DSM 14696
Corallococcus exiguus DSM 14696T
Corallococcus coralloides DSM 52499
Corallococcus coralloides DSM 51492
Corallococcus coralloides DSM 51425
Corallococcus coralloides DSM 51307
Corallococcus coralloides DSM 52502
Corallococcus coralloides DSM 52497
Corallococcus coralloides DSM 2259
Corallococcus coralloides NBRC 100086
Corallococcus coralloides DSM 51427
Corallococcus coralloides DSM 51379
Corallococcus coralloides DSM 51651
Corallococcus coralloides DSM 51682
Corallococcus coralloides DSM 51359
Corallococcus coralloides DSM 51557
Corallococcus coralloides DSM 51548
Corallococcus coralloides DSM 51547
Corallococcus coralloides DSM 51639
Corallococcus coralloides DSM 51620
Corallococcus coralloides DSM 51643
Corallococcus coralloides DSM 51298
Corallococcus coralloides DSM 52500
Corallococcus coralloides DSM 52501
Corallococcus coralloides DSM 51625
Corallococcus coralloides DSM 51619
Corallococcus coralloides DSM 51435
Corallococcus coralloides DSM 51301
Corallococcus coralloides DSM 51615
Corallococcus coralloides DSM 51433
Corallococcus coralloides DSM 51408
Corallococcus coralloides DSM 51461
Corallococcus coralloides DSM 51434
Myxococcus xanthus DSM 16526
Myxococcus macrosporus DSM 14697
Myxococcus stipitatus DSM 14675
Myxococcus fulvus DSM 16525
Corallococcus coralloides DSM 52496
Corallococcus coralloides DSM 52498
Pyxidicoccus fallax DSM 14698
Archangium gephyra DSM 2261
Vitiosangium subalbum MCy 10944
Polyangium cellulosum So ce90
