# Supplementary figures and images for: Genome Sequencing and Pan-Genome Analysis of 23 Corallococcus spp. Strains Reveal Unexpected Diversity, With Particular Plasticity of Predatory Gene Sets
Source: Front Microbiol. 2018 Dec 19;9:3187. doi: 10.3389/fmicb.2018.03187 (PMC6306037; doi:10.3389/fmicb.2018.03187)

## Slide 1
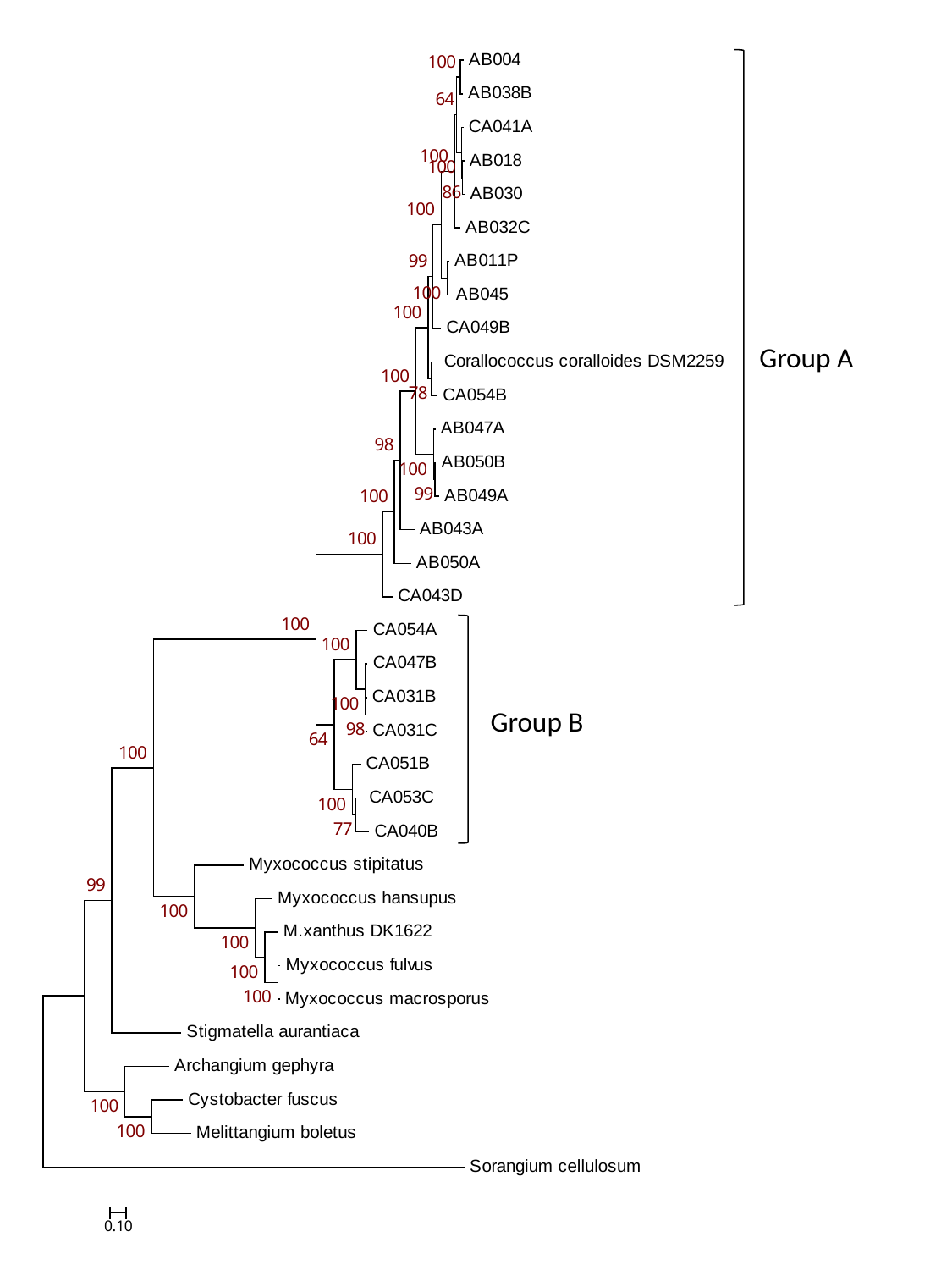

Group A
Group B

Supplement: Figure S2 — PhyloPhlAn phylogenetic tree of 400 gene sequences from the 24 genome-sequenced Corallococcus strains. [file Presentation_2.PPTX]

## Slide 1
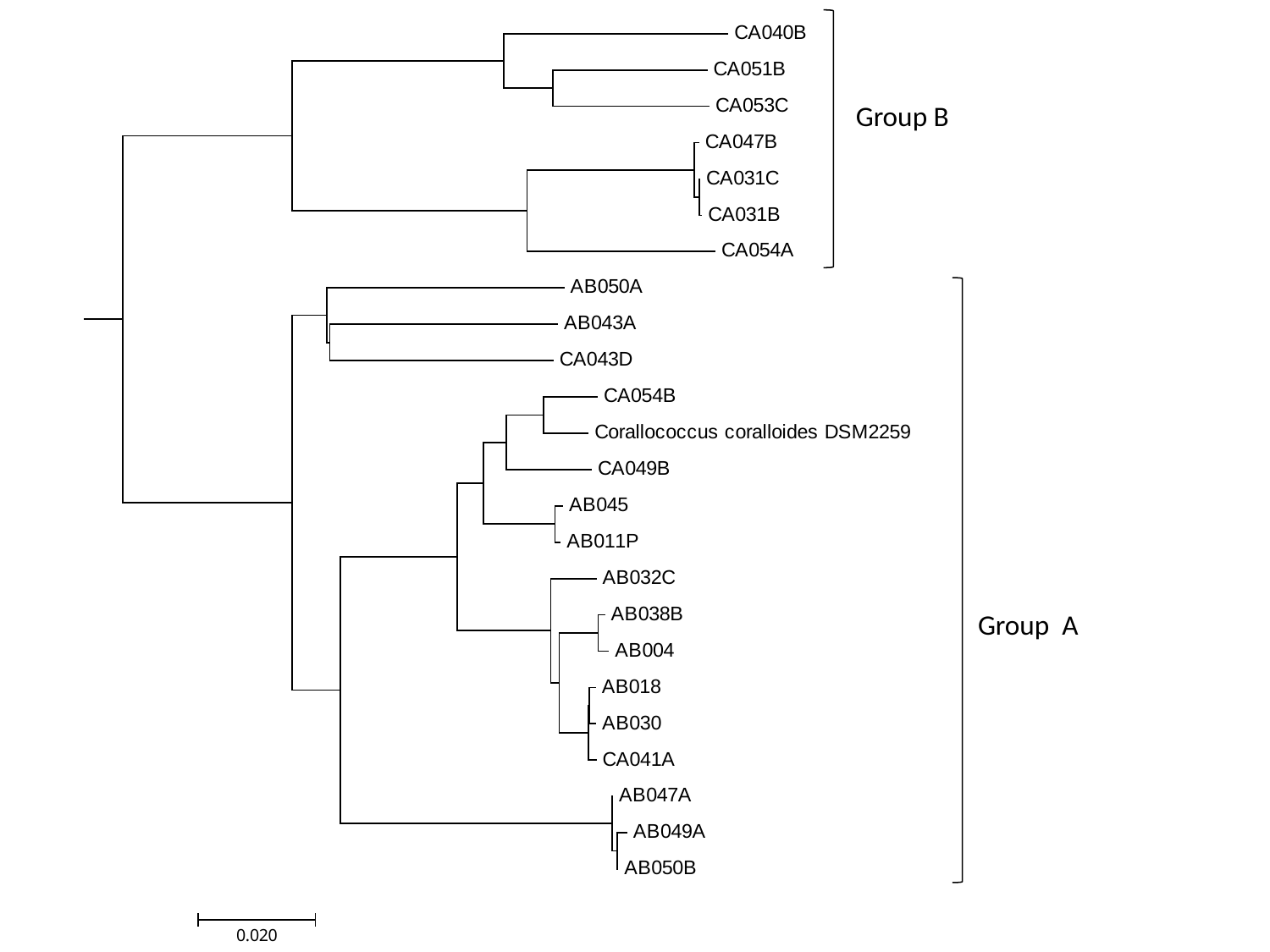

Group B
Group A

Supplement: Figure S3 — OrthoFinder tree based on the presence/absence matrix of orthogroups amongst the 24 sequenced Corallococcus genomes. [file Presentation_3.PPTX]
